# Ethyl 2-[3-(3,5-Dinitrobenzo­yl)thio­ureido]benzoate

**DOI:** 10.1107/S1600536811012918

**Published:** 2011-04-13

**Authors:** Sohail Saeed, Naghmana Rashid, Moazzam H. Bhatti, Wing-Tak Wong

**Affiliations:** a Department of Chemistry, Research Complex, Allama Iqbal Open University, Islamabad 44000, Pakistan; b Department of Chemistry, The University of Hong Kong, Pokfulam Road, Pokfulam, Hong Kong SAR, People’s Republic of China

## Abstract

In the title compound, C_17_H_14_N_4_O_7_S, the dihedral angle between the two benzene rings is 9.04 (15)°. The centroid–centroid distance of 3.9825 (19) Å between nearly parallel benzene rings of adjacent mol­ecules suggests the existence of π-π stacking. Inter­molecular and intra-mol­ecular N—H⋯O hydrogen bonding is present in the structure. The eth­oxy group is disordered over two sets of sites with an occupancy ratio of 0.580 (15):0.420 (15). The crystal studied was an inversion twin.

## Related literature

For background to the chemistry of thiourea derivatives and their bioloical activity, and a related structure, see: Saeed *et al.* (2010 ▶).
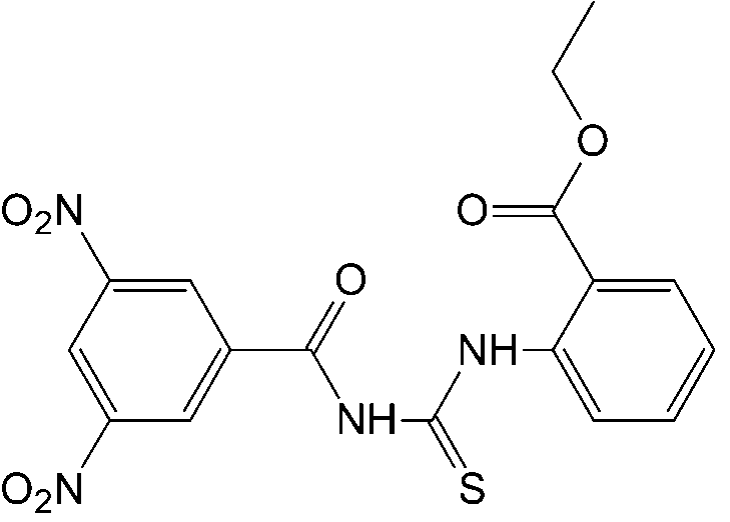


## Experimental

### 

#### Crystal data

C_17_H_14_N_4_O_7_S
*M* *_r_* = 418.38
Monoclinic, 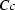

*a* = 11.7264 (19) Å
*b* = 16.617 (3) Å
*c* = 9.9630 (16) Å
β = 101.522 (2)°
*V* = 1902.3 (5) Å^3^
*Z* = 4
Mo *K*α radiation
μ = 0.22 mm^−1^
*T* = 299 K
0.27 × 0.16 × 0.08 mm

#### Data collection

Bruker SMART 1000 CCD diffractometer
Absorption correction: multi-scan (*SADABS*; Sheldrick, 2004 ▶) *T*_min_ = 0.943, *T*_max_ = 0.983
4944 measured reflections
3011 independent reflections
2727 reflections with *I* > 2σ(*I*)
*R*_int_ = 0.014

#### Refinement

*R*[*F* ^2^ > 2σ(*F* ^2^)] = 0.037
*wR*(*F* ^2^) = 0.095
*S* = 1.10
3011 reflections
301 parameters
42 restraints
H atoms treated by a mixture of independent and constrained refinement
Δρ_max_ = 0.22 e Å^−3^
Δρ_min_ = −0.24 e Å^−3^
Absolute structure: Flack (1983 ▶), 1340 Friedel pairs
Flack parameter: 0.39 (8)

### 

Data collection: *SMART* (Bruker, 1998 ▶); cell refinement: *SAINT* (Bruker, 2006 ▶); data reduction: *SAINT*; program(s) used to solve structure: *SHELXS97* (Sheldrick, 2008 ▶); program(s) used to refine structure: *SHELXL97* (Sheldrick, 2008 ▶); molecular graphics: *ORTEPII* (Johnson, 1976 ▶) and *Mercury* (Macrae *et al.*, 2008 ▶); software used to prepare material for publication: *SHELXL97*.

## Supplementary Material

Crystal structure: contains datablocks global, I. DOI: 10.1107/S1600536811012918/xu5177sup1.cif

Structure factors: contains datablocks I. DOI: 10.1107/S1600536811012918/xu5177Isup2.hkl

Additional supplementary materials:  crystallographic information; 3D view; checkCIF report

## Figures and Tables

**Table 1:** Hydrogen-bond geometry (Å, °)

*D*—H⋯*A*	*D*—H	H⋯*A*	*D*⋯*A*	*D*—H⋯*A*
N1—H1⋯O2	0.91 (3)	1.95 (3)	2.672 (3)	134 (2)
N1—H1⋯O3	0.91 (3)	2.01 (3)	2.700 (3)	131 (2)
N2—H2⋯O3^i^	0.77 (3)	2.32 (3)	3.086 (3)	172 (2)

Symmetry code: (i) 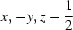.

**Fig. 1.**
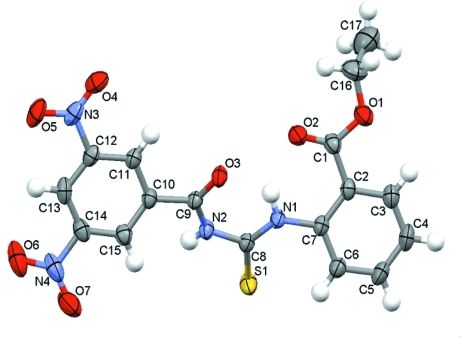
The ORTEP plot of the compound was shown at 50% probability thermal ellipsoids with the atom numbering scheme (only the major component was shown).

**Fig. 2.**
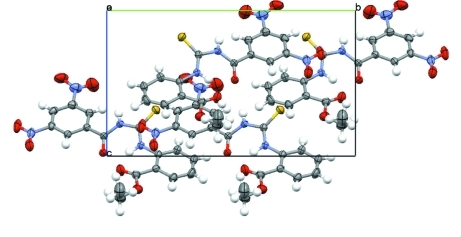
Packing diagram of the title compound viewed down the a axis.

## supplementary crystallographic information

### Comment

The background to this study has been set in our previous work on the structural chemistry of *N*,*N*'-disubstituted thiourea (Saeed *et al.*, 2010). Herein, as a continuation of these studies, the structure of the title compound, (I), C_17_H_14_N_4_O_7_S, is described.

The compound is slightly twisted. The nitro groups are 3.9 (5)° and 17 (1)° from the phenyl ring plane of C10—C15. The thiourea plane is making a dihedral angle of 5.3 (2)° with the amido group and makes a dihedral angle of 31.35 (17)° with the phenyl ring plane of C2—C7.

There are inter-molecular N—H···O H-bond interactions which link the molecules to form 1-D chain in the crystal lattice. There are also weak π···π between neighbouring rings in the crystal lattice.

### Experimental

A solution of 3,5-dinitrobenzoyl chloride (0.01 mol) in anhydrous acetone (75 ml) and 3% tetrabutylammonium bromide (TBAB) as a phase-transfer catalyst (PTC) in anhydrous acetone was added dropwise to a suspension of dry potassium thiocyanate (0.01 mol) in acetone (50 ml) and the reaction mixture was refluxed for 50 min. After cooling to room temperature, a solution of ethyl-orthoamino benzoate (0.01 mol) in anhydrous acetone (25 ml) was added dropwise and the resulting mixture refluxed for 3 h. Hydrochloric acid (0.1 N, 300 ml) was added, and the solution was filtered. The solid product was washed with water and purified by re-crystallization from ethyl acetate.

### Refinement

N-bound H-atoms were located in a difference Fourier map and refined isotropically. Other H atoms were placed at geometrical positions with C—H = 0.93–0.97 Å and refined using riding model with *U*_iso_(H) =1.2*U*_eq_(C). The ethoxy group is disordered over two sites, the occupancy ratio was refined to 0.580 (15):0.420 (15). Distance and displacement restraints were used for the disordered components.

### Crystal data

**Table:**

C_17_H_14_N_4_O_7_S	*F*(000) = 864
*M**_r_* = 418.38	*D*_x_ = 1.461 Mg m^−^^3^
Monoclinic, *C**c*	Mo *K*α radiation, λ = 0.71073 Å
Hall symbol: C -2yc	Cell parameters from 6458 reflections
*a* = 11.7264 (19) Å	θ = 2.1–25.0°
*b* = 16.617 (3) Å	µ = 0.22 mm^−^^1^
*c* = 9.9630 (16) Å	*T* = 299 K
β = 101.522 (2)°	Block, yellow
*V* = 1902.3 (5) Å^3^	0.27 × 0.16 × 0.08 mm
*Z* = 4	

### Data collection

**Table:**

Bruker SMART 1000 CCD diffractometer	3011 independent reflections
Radiation source: fine-focus sealed tube	2727 reflections with *I* > 2σ(*I*)
graphite	*R*_int_ = 0.014
ω and φ scans	θ_max_ = 25.0°, θ_min_ = 2.7°
Absorption correction: multi-scan (*SADABS*; Sheldrick, 2004)	*h* = −13→13
*T*_min_ = 0.943, *T*_max_ = 0.983	*k* = −19→15
4944 measured reflections	*l* = −11→11

### Refinement

**Table:**

Refinement on *F*^2^	Secondary atom site location: difference Fourier map
Least-squares matrix: full	Hydrogen site location: inferred from neighbouring sites
*R*[*F*^2^ > 2σ(*F*^2^)] = 0.037	H atoms treated by a mixture of independent and constrained refinement
*wR*(*F*^2^) = 0.095	*w* = 1/[σ^2^(*F*_o_^2^) + (0.0536*P*)^2^ + 0.3273*P*] where *P* = (*F*_o_^2^ + 2*F*_c_^2^)/3
*S* = 1.10	(Δ/σ)_max_ < 0.001
3011 reflections	Δρ_max_ = 0.22 e Å^−^^3^
301 parameters	Δρ_min_ = −0.24 e Å^−^^3^
42 restraints	Absolute structure: Flack (1983), 1340 Friedel pairs
Primary atom site location: structure-invariant direct methods	Flack parameter: 0.39 (8)

### Special details

**Table:**

Geometry. All e.s.d.'s (except the e.s.d. in the dihedral angle between two l.s. planes) are estimated using the full covariance matrix. The cell e.s.d.'s are taken into account individually in the estimation of e.s.d.'s in distances, angles and torsion angles; correlations between e.s.d.'s in cell parameters are only used when they are defined by crystal symmetry. An approximate (isotropic) treatment of cell e.s.d.'s is used for estimating e.s.d.'s involving l.s. planes.
Refinement. Refinement of *F*^2^ against ALL reflections. The weighted *R*-factor *wR* and goodness of fit *S* are based on *F*^2^, conventional *R*-factors *R* are based on *F*, with *F* set to zero for negative *F*^2^. The threshold expression of *F*^2^ > σ(*F*^2^) is used only for calculating *R*-factors(gt) *etc*. and is not relevant to the choice of reflections for refinement. *R*-factors based on *F*^2^ are statistically about twice as large as those based on *F*, and *R*- factors based on ALL data will be even larger.

### Fractional atomic coordinates and isotropic or equivalent isotropic displacement parameters (Å^2^)

**Table:**

	*x*	*y*	*z*	*U*_iso_*/*U*_eq_	Occ. (<1)
S1	0.94150 (8)	0.19793 (4)	0.68183 (8)	0.0759 (3)	
O2	0.8103 (2)	0.05570 (13)	1.0967 (3)	0.0866 (7)	
O3	0.99391 (19)	−0.01499 (10)	0.97679 (18)	0.0663 (5)	
O4	1.0187 (3)	−0.31100 (14)	0.9255 (4)	0.1022 (8)	
O5	1.1263 (3)	−0.35915 (14)	0.7937 (3)	0.1095 (10)	
O6	1.2496 (4)	−0.1832 (2)	0.4646 (3)	0.1355 (14)	
O7	1.2710 (4)	−0.0571 (3)	0.5114 (4)	0.1459 (15)	
N1	0.9176 (2)	0.13747 (12)	0.9269 (2)	0.0543 (5)	
H1	0.911 (2)	0.0912 (17)	0.974 (3)	0.064 (8)*	
H2	0.997 (2)	0.0472 (15)	0.704 (3)	0.045 (7)*	
N2	0.9902 (2)	0.05353 (12)	0.7789 (2)	0.0555 (5)	
N3	1.0798 (3)	−0.30348 (14)	0.8423 (3)	0.0773 (8)	
N4	1.2402 (3)	−0.1244 (3)	0.5338 (3)	0.0976 (10)	
C1	0.7736 (3)	0.11786 (17)	1.1281 (3)	0.0647 (7)	
C2	0.8103 (2)	0.19844 (15)	1.0865 (3)	0.0563 (6)	
C3	0.7770 (3)	0.26756 (18)	1.1485 (3)	0.0713 (8)	
H3	0.7296	0.2626	1.2125	0.086*	
C4	0.8124 (4)	0.3418 (2)	1.1176 (4)	0.0842 (10)	
H4	0.7885	0.3870	1.1596	0.101*	
C5	0.8830 (3)	0.35007 (17)	1.0248 (3)	0.0782 (9)	
H5	0.9080	0.4010	1.0051	0.094*	
C6	0.9179 (3)	0.28322 (16)	0.9596 (3)	0.0657 (7)	
H6	0.9662	0.2896	0.8968	0.079*	
C7	0.8809 (2)	0.20717 (14)	0.9882 (2)	0.0531 (6)	
C8	0.9476 (2)	0.13033 (14)	0.8043 (3)	0.0525 (6)	
C9	1.0156 (2)	−0.01157 (13)	0.8622 (2)	0.0518 (6)	
C10	1.0713 (2)	−0.08065 (14)	0.8043 (3)	0.0539 (6)	
C11	1.0528 (2)	−0.15737 (14)	0.8509 (3)	0.0555 (6)	
H11	1.0103	−0.1650	0.9193	0.067*	
C12	1.0991 (2)	−0.22190 (15)	0.7933 (3)	0.0595 (7)	
C13	1.1609 (3)	−0.21360 (18)	0.6905 (3)	0.0695 (8)	
H13	1.1890	−0.2582	0.6508	0.083*	
C14	1.1797 (3)	−0.1366 (2)	0.6489 (3)	0.0677 (8)	
C15	1.1379 (3)	−0.07002 (16)	0.7053 (3)	0.0618 (7)	
H15	1.1542	−0.0186	0.6775	0.074*	
O1	0.7136 (10)	0.1247 (5)	1.2262 (9)	0.083 (2)	0.580 (15)
C16	0.6730 (10)	0.0532 (7)	1.2838 (13)	0.113 (4)	0.580 (15)
H16A	0.6931	0.0548	1.3830	0.135*	0.580 (15)
H16B	0.7077	0.0056	1.2523	0.135*	0.580 (15)
C17	0.5404 (10)	0.0522 (6)	1.2345 (19)	0.161 (6)	0.580 (15)
H17A	0.5070	0.0125	1.2851	0.193*	0.580 (15)
H17B	0.5219	0.0392	1.1387	0.193*	0.580 (15)
H17C	0.5093	0.1042	1.2489	0.193*	0.580 (15)
O1'	0.6758 (12)	0.1178 (7)	1.1805 (14)	0.085 (3)	0.420 (15)
C16'	0.6436 (15)	0.0411 (7)	1.2287 (14)	0.084 (4)	0.420 (15)
H16C	0.7119	0.0073	1.2549	0.101*	0.420 (15)
H16D	0.5889	0.0139	1.1571	0.101*	0.420 (15)
C17'	0.5883 (19)	0.0568 (8)	1.3511 (18)	0.134 (6)	0.420 (15)
H17D	0.5738	0.0065	1.3920	0.161*	0.420 (15)
H17E	0.5162	0.0850	1.3219	0.161*	0.420 (15)
H17F	0.6399	0.0888	1.4169	0.161*	0.420 (15)

### Atomic displacement parameters (Å^2^)

**Table:**

	*U*^11^	*U*^22^	*U*^33^	*U*^12^	*U*^13^	*U*^23^
S1	0.1117 (6)	0.0590 (4)	0.0655 (4)	0.0263 (4)	0.0381 (4)	0.0158 (3)
O2	0.1105 (17)	0.0589 (12)	0.1056 (17)	0.0112 (11)	0.0580 (14)	0.0120 (11)
O3	0.1054 (16)	0.0478 (10)	0.0498 (10)	0.0112 (9)	0.0252 (10)	−0.0004 (7)
O4	0.131 (2)	0.0570 (13)	0.122 (2)	−0.0046 (13)	0.031 (2)	0.0083 (13)
O5	0.118 (2)	0.0524 (12)	0.153 (3)	0.0257 (13)	0.0147 (19)	−0.0192 (14)
O6	0.172 (3)	0.163 (3)	0.0823 (18)	0.073 (3)	0.052 (2)	−0.0065 (18)
O7	0.179 (4)	0.131 (3)	0.163 (3)	0.028 (3)	0.119 (3)	0.026 (2)
N1	0.0734 (15)	0.0413 (10)	0.0509 (12)	0.0056 (9)	0.0185 (10)	−0.0003 (8)
N2	0.0776 (15)	0.0480 (11)	0.0434 (12)	0.0094 (10)	0.0178 (11)	−0.0029 (9)
N3	0.0820 (18)	0.0479 (13)	0.092 (2)	0.0105 (12)	−0.0056 (16)	−0.0079 (13)
N4	0.099 (2)	0.123 (3)	0.0795 (19)	0.049 (2)	0.0396 (17)	0.0068 (19)
C1	0.076 (2)	0.0658 (17)	0.0559 (15)	0.0108 (14)	0.0209 (14)	0.0059 (12)
C2	0.0635 (17)	0.0569 (14)	0.0459 (13)	0.0114 (11)	0.0046 (12)	−0.0053 (10)
C3	0.081 (2)	0.0679 (18)	0.0663 (18)	0.0144 (14)	0.0178 (16)	−0.0096 (14)
C4	0.103 (3)	0.0639 (19)	0.086 (2)	0.0187 (17)	0.0186 (19)	−0.0250 (16)
C5	0.103 (2)	0.0453 (14)	0.084 (2)	0.0031 (14)	0.0121 (18)	−0.0134 (13)
C6	0.080 (2)	0.0526 (14)	0.0644 (17)	−0.0012 (12)	0.0144 (15)	−0.0065 (12)
C7	0.0631 (16)	0.0459 (12)	0.0478 (13)	0.0070 (10)	0.0049 (12)	−0.0029 (10)
C8	0.0577 (15)	0.0476 (13)	0.0520 (13)	0.0034 (10)	0.0100 (11)	−0.0010 (10)
C9	0.0643 (17)	0.0453 (13)	0.0462 (14)	0.0029 (10)	0.0119 (12)	−0.0023 (10)
C10	0.0649 (17)	0.0507 (13)	0.0445 (12)	0.0062 (11)	0.0069 (12)	−0.0030 (10)
C11	0.0637 (17)	0.0486 (13)	0.0516 (13)	0.0047 (11)	0.0056 (12)	−0.0062 (10)
C12	0.0622 (17)	0.0462 (13)	0.0641 (17)	0.0098 (11)	−0.0015 (14)	−0.0033 (11)
C13	0.0718 (19)	0.0740 (18)	0.0574 (16)	0.0290 (14)	0.0001 (14)	−0.0162 (14)
C14	0.0655 (18)	0.082 (2)	0.0568 (16)	0.0240 (14)	0.0151 (14)	0.0023 (14)
C15	0.0686 (18)	0.0592 (14)	0.0581 (15)	0.0113 (13)	0.0138 (13)	0.0029 (12)
O1	0.103 (6)	0.083 (3)	0.075 (4)	0.006 (3)	0.043 (4)	0.008 (3)
C16	0.130 (8)	0.116 (7)	0.104 (7)	−0.012 (5)	0.053 (6)	0.018 (5)
C17	0.191 (12)	0.086 (5)	0.212 (14)	−0.002 (6)	0.056 (10)	0.002 (7)
O1'	0.087 (6)	0.081 (4)	0.093 (7)	0.005 (4)	0.035 (5)	−0.008 (4)
C16'	0.090 (7)	0.070 (5)	0.108 (8)	−0.013 (4)	0.055 (6)	0.007 (5)
C17'	0.209 (14)	0.092 (7)	0.135 (10)	0.039 (8)	0.116 (10)	0.024 (7)

### Geometric parameters (Å, °)

**Table:**

S1—C8	1.649 (2)	C6—C7	1.385 (4)
O2—C1	1.185 (3)	C6—H6	0.9300
O3—C9	1.219 (3)	C9—C10	1.493 (3)
O4—N3	1.205 (4)	C10—C15	1.386 (4)
O5—N3	1.222 (4)	C10—C11	1.388 (3)
O6—N4	1.213 (4)	C11—C12	1.377 (4)
O7—N4	1.210 (5)	C11—H11	0.9300
N1—C8	1.342 (3)	C12—C13	1.375 (4)
N1—C7	1.416 (3)	C13—C14	1.376 (5)
N1—H1	0.91 (3)	C13—H13	0.9300
N2—C9	1.360 (3)	C14—C15	1.375 (4)
N2—C8	1.412 (3)	C15—H15	0.9300
N2—H2	0.77 (3)	O1—C16	1.442 (8)
N3—C12	1.474 (4)	C16—C17	1.535 (9)
N4—C14	1.478 (4)	C16—H16A	0.9700
C1—O1	1.318 (10)	C16—H16B	0.9700
C1—O1'	1.351 (14)	C17—H17A	0.9600
C1—C2	1.490 (4)	C17—H17B	0.9600
C2—C3	1.396 (4)	C17—H17C	0.9600
C2—C7	1.410 (4)	O1'—C16'	1.440 (9)
C3—C4	1.356 (5)	C16'—C17'	1.514 (9)
C3—H3	0.9300	C16'—H16C	0.9700
C4—C5	1.365 (5)	C16'—H16D	0.9700
C4—H4	0.9300	C17'—H17D	0.9600
C5—C6	1.389 (4)	C17'—H17E	0.9600
C5—H5	0.9300	C17'—H17F	0.9600
			
C8—N1—C7	128.5 (2)	N2—C9—C10	115.8 (2)
C8—N1—H1	117.1 (18)	C15—C10—C11	120.2 (2)
C7—N1—H1	114.0 (18)	C15—C10—C9	122.0 (2)
C9—N2—C8	130.7 (2)	C11—C10—C9	117.9 (2)
C9—N2—H2	115.4 (18)	C12—C11—C10	118.4 (3)
C8—N2—H2	113.8 (19)	C12—C11—H11	120.8
O4—N3—O5	124.6 (3)	C10—C11—H11	120.8
O4—N3—C12	118.4 (2)	C13—C12—C11	122.8 (3)
O5—N3—C12	117.1 (3)	C13—C12—N3	118.5 (2)
O7—N4—O6	125.1 (4)	C11—C12—N3	118.6 (3)
O7—N4—C14	118.6 (3)	C12—C13—C14	117.2 (2)
O6—N4—C14	116.2 (4)	C12—C13—H13	121.4
O2—C1—O1	123.2 (5)	C14—C13—H13	121.4
O2—C1—O1'	118.9 (6)	C15—C14—C13	122.3 (3)
O2—C1—C2	124.7 (3)	C15—C14—N4	118.1 (3)
O1—C1—C2	110.8 (4)	C13—C14—N4	119.5 (3)
O1'—C1—C2	115.0 (5)	C14—C15—C10	119.0 (3)
C3—C2—C7	118.5 (2)	C14—C15—H15	120.5
C3—C2—C1	119.6 (3)	C10—C15—H15	120.5
C7—C2—C1	121.9 (2)	C1—O1—C16	119.4 (9)
C4—C3—C2	121.5 (3)	O1—C16—C17	106.0 (9)
C4—C3—H3	119.2	O1—C16—H16A	110.5
C2—C3—H3	119.2	C17—C16—H16A	110.5
C3—C4—C5	120.0 (3)	O1—C16—H16B	110.5
C3—C4—H4	120.0	C17—C16—H16B	110.5
C5—C4—H4	120.0	H16A—C16—H16B	108.7
C4—C5—C6	120.7 (3)	C1—O1'—C16'	115.3 (10)
C4—C5—H5	119.6	O1'—C16'—C17'	107.4 (10)
C6—C5—H5	119.6	O1'—C16'—H16C	110.2
C7—C6—C5	120.0 (3)	C17'—C16'—H16C	110.2
C7—C6—H6	120.0	O1'—C16'—H16D	110.2
C5—C6—H6	120.0	C17'—C16'—H16D	110.2
C6—C7—C2	119.3 (2)	H16C—C16'—H16D	108.5
C6—C7—N1	121.5 (2)	C16'—C17'—H17D	109.5
C2—C7—N1	119.1 (2)	C16'—C17'—H17E	109.5
N1—C8—N2	114.2 (2)	H17D—C17'—H17E	109.5
N1—C8—S1	129.22 (19)	C16'—C17'—H17F	109.5
N2—C8—S1	116.62 (19)	H17D—C17'—H17F	109.5
O3—C9—N2	123.2 (2)	H17E—C17'—H17F	109.5
O3—C9—C10	121.0 (2)		

### Hydrogen-bond geometry (Å, °)

**Table:**

*D*—H···*A*	*D*—H	H···*A*	*D*···*A*	*D*—H···*A*
N1—H1···O2	0.91 (3)	1.95 (3)	2.672 (3)	134 (2)
N1—H1···O3	0.91 (3)	2.01 (3)	2.700 (3)	131 (2)
N2—H2···O3^i^	0.77 (3)	2.32 (3)	3.086 (3)	172 (2)

Symmetry codes: (i) *x*, −*y*, *z*−1/2.
